# Affective Switch Associated With Oral, Low Dose Ketamine Treatment in a Patient With Treatment Resistant Bipolar I Depression. Case Report and Literature Review

**DOI:** 10.3389/fpsyt.2020.00516

**Published:** 2020-06-03

**Authors:** Alina Wilkowska, Łukasz Szałach, Jakub Słupski, Aleksandra Wielewicka, Małgorzata Czarnota, Maria Gałuszko-Węgielnik, Mariusz S. Wiglusz, Wiesław J. Cubała

**Affiliations:** Department of Psychiatry, Medical University of Gdansk, Gdansk, Poland

**Keywords:** ketamine, oral and subanaesthetic, affective switch, bipolar I depression, treatment resistance

## Abstract

There is a growing evidence for the rapid and robust antidepressive effect of ketamine in unipolar and bipolar treatment resistant depression although evidence for the risk of affective switch is still limited. This case presents bipolar I disorder patient with treatment resistant depressive episode experiencing a switch to manic episode with mixed features shortly after receiving eight subanaesthetic doses of oral ketamine as an add-on treatment preceded by 2-day period of manic symptoms.

## Background

Bipolar disorder (BPD) is one of the most severe and lethal of all psychiatric disorders (1, 2). There is an unmet need for more effective treatment strategies in bipolar depression (3). Mounting evidence supports effectiveness of single low dose ketamine in treatment resistant cases of bipolar depression (4–6). Data on the effectiveness and risk of polarity switch in bipolar patients treated with ketamine is still insufficient. This case report presents a 28-year-old BP I patient with severe depressive episode experiencing a switch to mania with mixed features after eight doses of oral ketamine. To our knowledge this is the first report of affective switch associated with oral ketamine.

## Case Report

A 28-year-old Caucasian female with bipolar I disorder diagnosed 4 years before, was hospitalized in an inpatient psychiatry clinic due to treatment-resistant depressive episode with extensive suicidal thoughts. Mental status has been deteriorating for 4 months before admission. During her illness, the patient was hospitalized five times due to depressive episodes and had two manic episodes without hospitalization. The patient had been treated with ketamine twice, first during previous hospitalization (IV) and second time during described stay (oral).

### Previous Hospitalization

One year before she was hospitalized in the same facility due to severe treatment resistant depressive episode and despite treatment modifications she did not achieve remission, thus she was offered ketamine treatment. A dosage of 0.5mg/kg ketamine hydrochloride intravenous infusion over a period of 40 min was given two times per week for a period of 4 weeks (eight times in total) as add-on treatment to standard of care. After the ketamine administration period, an intermittent mood improvement lasting 1 week has been observed (six points reduction in MADRS score)—no manic symptoms appeared. Further pharmacological modifications were made—after 6-month hospitalization patient achieved partial remission and was discharged on lamotrigine 400 mg/day, lithium carbonate 750 mg/day, clozapine 100 mg/day, and topiramate 400 mg/day.

### Present Hospitalization

On admission the patient presented decreased mood, decreased energy, suicidal thoughts, feeling of constant inner tension, difficulties concentrating, withdrawal from social interactions, sleeping difficulties. Somatic causes were excluded after physical examination, neurological examination, and laboratory tests (blood morphology, electrolytes, kidney and liver profile, TSH, FT4, CRP, B12, folate levels, urine test, toxicology) which turned out normal.

During the time since her previous hospitalization, the treatment has been modified in the outpatient care—clozapine has been discontinued, the dose of topiramate has been reduced to 100 mg/day, bupropion 300 mg/day, and chlorprothixene 60 mg/day have been added. Lithium serum level was 0,87 mmol/L on dose of 1,000 mg/day. In the EEG record slow basic activity and slow waves in the front-temporo-parietal region on the left side were described; the MRI scan did not reveal any pathological changes. The pharmacological changes listed above did not cause any improvement; for this reason the patient was qualified for readministration of ketamine, this time in oral form.

The dose of ketamine administered orally was 2.5 mg/kg. During the first 10 min half of the dosage was administered and in the next 30 min the remaining part was sipped. Ketamine was administered twice weekly during 4 weeks. After 2nd week of ketamine administration the daily dose of lithium was reduced due to its increased serum level (1.42 mmol/L) to 750mg/day which resulted in 0.74 mmol/L serum concentration. During the third week of oral ketamine administration period, manic symptoms were observed. The patient presented increased sex drive, elevated psychomotor activity, racing thoughts, irritability, problems falling asleep and total sleep reduction. She spent a significant amount of money on online shopping, cleaned her locker several times, and became talkative. The symptoms were present for 2 days; after that time depressive symptoms returned with additional appetite increase. Thus decision for continuation of ketamine treatment was made. One day after the last dose of ketamine, the patient presented manic symptoms again. She had intermittent periods of elevated mood, her energy was constantly increased, she presented maladaptive affect regulation, decreased need for sleep, increased sex drive, she became talkative, dressed inappropriately, had increased appetite. The patient demonstrated psychomotor agitation with accompanying suicidal thoughts, lasting for 1 week. Depressive symptoms were still present, but manic symptoms clearly dominated. No psychotic symptoms were present. Drug screen was negative. One week after finishing ketamine administration lithium dose was increased again up to 1,000 mg/day reaching serum level 1.11 mmol/L.

**Figure 1:**
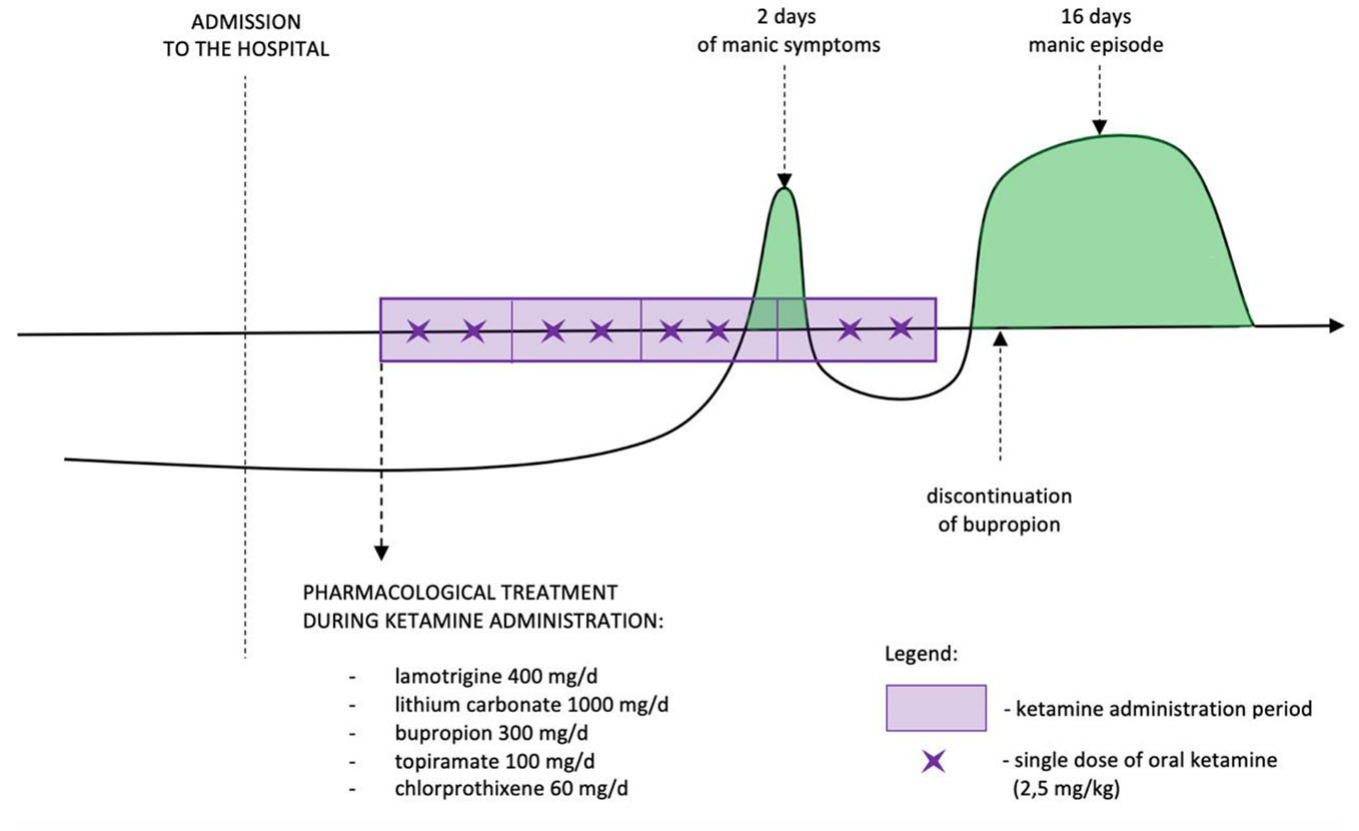
Treatment timeline.

The symptoms resolved after a period of 16 days. During first three doses of ketamine administration patient presented sedation, dizziness, and some dissociative symptoms. The symptoms were of moderate intense and resolved during next 40 min after the last sip of oral ketamine. No other significant adverse events were observed. Treatment timeline is presented in **Figure 1**.

### Safety and Psychometric Scales

Both protocols, intravenous and oral, included recording of basic life parameters such as heart rate, arterial pressure, oxygen saturation level, respiratory rate and body temperature measured every 15 min. In both protocols psychometric assessment included: Montgomery Åsberg Depression Rating Scale structured interview (MADRS-Sigma) and Young Mania Rating Scale (YMRS) completed before IV and oral administrations and after 3rd, 5th, and 7th dose as well as 1 week after finishing the treatment.

### Oral Ketamine

The oral route of administration is probably the least costly and most acceptable way to administer ketamine in patients with depression. The existing literature on oral ketamine suggests that oral doses should range from 2–3 mg/kg. It is approximately the dose of 0.5 mg/kg IV multiplied by the oral bioavailability correction factor which is 4–5. This factor is a result of high first-pass metabolism (7). It is also the dose most commonly used in the oral ketamine study by Hartberg (8). Nevertheless it is worth noticing that off-label use of oral ketamine is based on very limited research and no dose-ranging study has been published so far. Additionally antidepressive effect and possible adverse effects of norketamine—the main metabolite of ketamine administered orally are presently unknown although no serious adverse events have been reported so far (7).

The case report is a part of the naturalistic ketamine trial, approved by the bioethical regulatory of the institution, NKBBN/172/2017; 172-674/2019, NCT04226963. The patient was informed about potential risks and limitations as well as reasonable expectations of ketamine treatment for depression and consent for treatment and gave an informed consent for the participation in the trial. The previously mentioned cases are presented according to guidelines for disguising case material.

The MADRS and YMRS scores and concomitant medications are summarized in **Table 1**.

**Table 1:** Ketamine treatment, concomitant medications, and psychometrics.

	Period of ketamine treatment	Route of administration	Administration time	Number of doses received	Ketamine dosage	Bipolar Disorder phase before initiation	Medications taken during ketamine treatment	MADRS score					YMRS score				
First administration	4 weeks	i.v. infusions	40 min	8	0.5 mg/kg	Depressive episode	-valproate 1,300 mg/day -lithium carbonate 1,000 mg/day -lamotrigine 200 mg/day	0	3rd dose	5th dose	7th dose	end	0	3rd dose	5th dose	7th dose	end
								33	39	40	36	27	2	0	0	0	0
Second administration	3 weeks	Oral	40 min	8	2.5 mg/kg	Depressive episode	- lithium carbonate 1,000 mg/day - bupropion 600 mg/day - lamotrigine 300 mg/day - chlorprotixene 60 mg/day - gabapentine 600 mg/day	0	3rd dose	5th dose	7th dose	end	0	3rd dose	5th dose	7th dose	end
								37	31	29	35	35	0	1	7	7	17

## Discussion

Presented case reports polarity switch in bipolar patient treated with eight doses of oral ketamine. Described symptoms reached the International Society for Bipolar Disorders Task Force criteria of a “likely” treatment-emergent affect switch (i.e., two manic symptoms lasting more than 50% of the day for 2 days, and a Young Mania Rating Scale (YMRS) greater than 12) (9).

To our knowledge, eight cases of affective switch associated to ketamine have been described so far. None of them however concerned oral administration. The first case report was criticized for the weak evidence of connection to ketamine and the possibility of misdiagnosis (10, 11). Following two cases reported manic symptoms after ketamine infusions for depression. The first case reported a patient with unipolar depression who was administered ketamine 0.3 mg/kg intramuscularly, three times over 6 days, and YMRS scores were 17, 18, and 20 after each injection. In this case, mania emerged in the absence of concomitant medications (12). The second BP I patient after single infusion of 0.5 mg/kg ketamine, developed elevated mood, hypersexuality, and excessive spending despite a therapeutic lithium level (13). The next case describes a patient with active substance use disorder who underwent surgical intervention (14). To reduce postoperative opioid use, ketamine was infused at 0.2 mg/kg/h over 4 days, and expansive affect, sleep-deprived energy enhancement, grandiosity, hypersexuality, and disinhibition occurred. The fifth case of reported ketamine-induced mania described a patient with Tourette syndrome and OCD who abused ketamine by inhaling (15). One case report describes a 17-year-old man with cerebral palsy experiencing hypomanic-like symptoms possibly connected to two ketamine administrations for sedation during dental procedures. Naranjo Adverse Drug Reaction Probability Scale, suggested a probable causal relationship in the described case (16). The next case presents a prolonged manic episode with psychotic symptoms in a young woman with unipolar depression, substance use disorder and CRPS (complex regional pain syndrome) treated with ketamine infusion 10, 15, and 20 mg/h, (her weight was 56 kg). Manic symptoms lasted for about 3 weeks, the pain completely resolved. For months prior to hospitalization she received ketamine treatment for 2 days in similar doses. No change in her mood was observed then (17). The most recent case presents a patient with possible genetic predisposition to BD who developed mania after receiving ketamine as a part of pain management and sedation (18). The above case reports are described in **Table 2**.

**Table 2:** Previous cases reporting affective switch associated to ketamine.

Author	patient	Diagnosis	Ketamine dose	Rout of administration	Type and length of switch and treatment	Concomitant medications
Ricke et al. (10)	F 42	reflex sympathetic dystrophy, opioid use disorder active, history of depression	(0.1–0.2 mg/kg/h) over 5 days.	i.v.	mania/hypomania 3 weeks quetiapine 200mg	duloxetine 20 mg/day, mirtazapine 45 mg/day quetiapine 100 mg/day
Banwari et al. (12)	M 52	unipolar depression	ketamine 0.3 mg/kg, three times over 6 days	i.m.	mania 2 weeks YMRS scores 17, 18, and 20 after each injection. Lithium carbonate 900 mg. lorazepam 2 mg	none
McInnes et al. (13)	F 44	bipolar I depression, substance use disorder (alcohol, cannabis active)	0.5 mg/kg over 40 min	i.v	mania 1 week followed by mixed state with suicidality lithium carbonate 900, bupropion XR	aripiprazole 10 mg, modafinil 100 mg, clonazepam 1 mg alternating with zolpidem 10 mg and lorazepam 0.5 mg, lithium carbonate 600 mg,
Nichols et al. (14)	M 27	severe chronic pain due to compartment syndrome, multiple substance use disorder active	0.2mg/kg/h he had received 19mg/kg cumulatively over 4 days.	i.v.	mania 3 weeks olanzapine 20/day lorazepam 4 mg/day	acetaminophen i.v. 3,000 mg/day, gabapentin 400 mg/day, hydromorphone Patient Controlled Analgesia (PCA) 0.4 mg i.v. every 10 min, up to 6 mg in 4 h (average of 30 mg per 24 h received, equivalent to 600 mg of morphine),
Lu et al. (15)	M 26	ketamine substance abuse 10–15 g/week for 12 months, Tourette syndrome, obsessive-compulsive disorder (OCD)	–	inhaled	manic-like symptoms after stopping ketamine use 5 months, diminished OCD symptoms, followed by depressive episode	
Marta et al. (16)	M 17	spastic cerebral palsy, dental decay	two ketamine administrations for anesthesia	i.v.	hypomania 1 week, followed by agitation lasting few months	gabapentin, risperidone, guanfacine, carbamazepine, clonazepam, lorazepam, oxybutynin, nitrofurantoin, omeprazole, polyethylene glycol
Mandyam and Ahuja (17)	F 30	complex regional pain syndrome (CRPS), unipolar depression, anxiety, three suicidal attempts, substance use disorder opioid remission, cannabies -active	0.18 mg/kg/h–0.45 mg/kg/h) for 4 days	i.v.	mania with psychotic symptoms few weeks, olanzapine, then quetiapine 600mg, duloxetine reduced to 20mg, pain completely resolved	duloxetine 60 mg
Allen et al. (18)		abdominal hernia fistulae need of surgery, History of post-partum depression and seasonal affective disorder (SAD). Her son had BD	postoperative pain management 0.1–0.15 mg/kg two or three times a day for 4 days total 520mg	i.v. boluses	mania about 10 days olanzapine 30 mg, 20 mg, and 5 mg	duloxetine 60mg discontinued 1 week prior to ketamine treatment, Propofol, Morphine,

It appears that some specific features predispose to manic switch associated with ketamine treatment. Mentioned cases report mainly subjects with substance use disorder, moreover in half of reported cases monoaminergic antidepressants were used. The patient described in this report was treated with mood stabilizers, but also received bupropion. According to one metanalysis bupropion, previously associated with low risk of polarity switch in bipolar disorder causes it as often as other antidepressants (19). Our observation and previous case reports suggest increased risk of switch associated to ketamine administered together with antidepressants. Although the neurobiological processes causing affective switch are still poorly understood there is evidence suggesting that genes regulating monoaminergic transmission, circadian rhythms and neurotrophic factors like BDNF may be responsible for increased risk of treatment-emergent switch (20). BDNF role in switch process is particularly interesting considering the evidence from animal studies showing increase after ketamine administration (21). Bipolar patients experiencing switches associated to treatment have more severe course of the disorder including cycle acceleration and increased suicide risk (22, 23). They also more often develop substance use disorder (24). It is possible that substance use disorders in bipolar patients predispose to switch, but this question is still to be answered.

Data from three studies of unipolar and bipolar patients with treatment resistant depression, randomized to subanaesthetic ketamine or placebo revealed transient mood elevation in 3 of 44 (7%) patients receiving placebo and 5 of 52 (10%) patients given ketamine, but none met the International Society for Bipolar Disorders Task Force criteria The authors concluded that the data did not support a persistent substance-induced syndrome, since the patients’ mood returned to baseline by the following day and without additional intervention (11).

Taken together it seems that affective switch is a possible complication of ketamine treatment and the risk might be increased in bipolar patients and patients with substance use disorder. Ketamine administered together with antidepressants likely predisposes to affective switch. It also seems that opioid treatment which is particularly important in case of patients with chronic pain can also increase the risk. Available, although limited data suggest that manic symptoms associated with ketamine can be effectively treated with olanzapine, quetiapine and lithium. We hypothesize that treating treatment resistant bipolar depression with ketamine should be accompanied by mood stabilizers and antidepressant should be avoided during this time. It also seems reasonable to conduct the drug screen before starting ketamine treatment. It is still unknown if the route of ketamine administration the level of ketamine metabolite—norketamine can be involved in the risk of affective switch. More studies are needed in order to discover neurobiological processes causing this phenomenon.

### Limitations

The case report does not reflect the wider population nor a causative relationship, and thus, replication in a proof of concept study is warranted in a larger population. Longer follow-up might be warranted.

## Conclusion

This case report suggests that polarity switch should be considered as a potential side effect while using ketamine for treatment-resistant bipolar depression. There is a significant need for further studies of safety and efficacy of oral and IV ketamine as add-on treatment in bipolar patients.

## Data Availability Statement

All datasets generated for this study are included in the article/supplementary material.

## Ethics Statement

The studies involving human participants were reviewed and approved by Independent Ethics Committee of the Medical University of Gdansk NKBBN/172/2017; 172-674/2019,NCT04226963. The patients/participants provided their written informed consent to participate in this study. Written informed consent was obtained from the individual(s) for the publication of any potentially identifiable images or data included in this article.

## Author Contributions

AW contributed to the manuscript draft and research on the topic. ŁS, MC, and AW contributed to the patient management and elaborated on patients’ medical records JS administered ketamine and evaluated the patient. MG-W directed the naturalistic case registry and elaborated on patients’ records. MW and WC conceptualized the study and corrected the manuscript.

## Funding

This paper was granted by the Medical University of Gdańsk, Poland (Grant No. ST-02-0039/07/221).

## Conflict of Interest

AW has received research support from Angelini, Biogen, Eli Lilly and Company, Janssen-Cilag, Lundbeck, Polpharma, Sanofi and Valeant. ŁS has received support from Angelini and +Pharma. JS has received research support from Actavis, Celon, Eli Lilly, Minerva Neurosciences, and Sunovion Pharmaceuticals; Speakers bureau: Promed, Teva, Valeant. MC has received support from Angelini, Janssen-Cilag and Adamed. MG-W has received research support from Alkermes, Biogen, Celon, Janssen, KCR, Minerva Neurosciences, Lilly, and Servier. MW has received research support from Alkermes, Auspex Pharmaceuticals, Biogen, Cephalon, Celon, Cortexyme, Eli Lilly, Ferrier, Forest Laboratories, GedeonRichter, GWPharmaceuticals, Janssen, Lundbeck, Orion, Otsuka, and Servier; Speakers bureau: Lundbeck, Servier. WC has received research support from Acadia, Actavis, Alkermes, Allergan, Apodemus, Auspex, Biogen, Bristol-Myers Squibb, Cephalon, Celon, Cortexyme, Eli Lilly, Ferrier, Forest Laboratories, Gedeon Richter, GW Pharmaceuticals, Janssen, KCR, Lundbeck, NIH, NeuroCog, Orion, Otsuka, Sanofi, and Servier; he has served on speakers bureaus for Adamed, Angelini, AstraZeneca, Bristol-Myers Squibb, Celon, GlaxoSmithKline, Janssen, Krka, Lekam, Lundbeck, Novartis, Orion, Pfizer, Polfa Tarchomin, Sanofi, Servier, and Zentiva; and he has served as a consultant for GW Pharmaceuticals, Janssen, KCR, Quintiles, and Roche.
